# A metabolic-radioimmune signature predicts therapy response and immune reprogramming in non-small cell lung cancer

**DOI:** 10.3389/fonc.2025.1693277

**Published:** 2025-11-11

**Authors:** Zihong Zhu, Yichen Zan, Mengqian Jiang, Ran Zhang, Dawei Chen, Guanglu Dong

**Affiliations:** 1 Department of Radiation Oncology, the Second Affiliated Hospital of Harbin Medical University, Harbin, Heilongjiang, China; 2 Department of Radiation Oncology and Shandong Provincial Key Laboratory of Precision Oncology, Shandong Cancer Hospital and Institute, Shandong First Medical University and Shandong Academy of Medical Sciences, Jinan, Shandong, China; 3 Harbin Medical University, Harbin, Heilongjiang, China; 4 School of Clinical Medicine, Shandong Second Medical University, Weifang, Shandong, China

**Keywords:** non-small cell lung cancer, metabolic reprogramming, radioresistance, tumorimmune microenvironment, radioresistance-related metabolic genes

## Abstract

**Objective:**

This study systematically investigates radiotherapy-induced metabolic remodeling across the TME, encompassing tumor cells, immune cells, and tumor-draining lymph nodes (TDLNs), and establishes a prognostic signature based on radioresistance-related metabolic genes (RRMGs) to optimize therapeutic stratification and radiosensitizer discovery.

**Methods:**

Bulk transcriptomic datasets of NSCLC tumor cells and tumor-draining TDLNs were systematically integrated, along with single-cell RNA-seq data from tumor tissues, to reconstruct metabolic flux maps using the single-cell Flux Estimation Analysis (scFEA) algorithm. WGCNA and Cox regression modeling of TCGA radiotherapy cohort were used to identify core RRMGs. A prognostic nomogram was developed using risk scores derived from these genes, while CIBERSORT and TIDE algorithms were used to evaluated TIME features and immunotherapy responses. Candidate radiosensitizing agents were predicted via the oncoPredict platform and validated by molecular docking, qRT-PCR and western blotting in radioresistant NSCLC cells.

**Results:**

Radiotherapy induced profound metabolic heterogeneity across the NSCLC TIME: Tumor cells and draining TDLNs exhibited suppressed tricarboxylic acid (TCA) cycle activity and N-glycan biosynthesis, while immune cells displayed upregulated serine metabolism alongside divergent shifts in lymphoid subsets. Seven RRMGs were identified as key prognostic determinants, including *PGD, IDH2, G6PD, ALDH3A1, UPP1, XYLT2, AACS*. The RRMG-based risk model robustly predicted poor overall survival (*HR* = 4.726, 95% *CI*: 2.154-10.371; *P*<0.001), with high predictive accuracy (AUC for 1-, 3-, and 5-year: 0.752, 0.778, and 0.879). High-risk patients demonstrated an immunosuppressive TIME marked by elevated tumor-promoting immune cell infiltration and TIDE scores. The model’s generalizability was verified in an independent radioimmunotherapy cohort (AUC: 0.618). Experimental validation revealed significant upregulation of high-risk RRMGs in radioresistant NSCLC cells. Ouabain and two novel compounds (BRD-K28456706, BRD-K42260513) were nominated as promising radiosensitizers.

**Conclusion:**

Radiotherapy-induced metabolic reprogramming in TIME drives resistance of NSCLC. The RRMG signature predicts radioimmunotherapy outcomes for patient stratification. Identifying ouabain and novel compounds highlights targeting metabolic vulnerabilities as a translatable strategy to overcome resistance.

## 1 Introduction

Lung cancer remains the leading cause of cancer-related mortality worldwide, with non-small cell lung cancer (NSCLC) accounting for the majority of cases. The high rates of recurrence and metastasis contribute to its poor prognosis (1). Radiotherapy, a cornerstone treatment for locally advanced NSCLC, exerts its antitumor effects by directly inducing DNA damage or indirectly generating reactive oxygen species (ROS) (2–4). In recent years, immunotherapy has revolutionized cancer therapies, and radiotherapy has been shown to synergize with immune checkpoint blockers (ICBs) by enhancing CD8+ T-cell infiltration and antigen presentation, among other mechanisms (5). The combination of these modalities holds promise for converting “cold tumors” into “hot tumors.” However, radioresistance remains a major cause of treatment failure, underscoring the urgent need to develop predictive systems for radiosensitivity. Conventional studies have primarily focused on unidimensional mechanisms, such as DNA repair and the tumor immune microenvironment, yet their clinical translation in sensitivity prediction remains limited.

Tumor metabolic reprogramming influences radiotherapy efficacy through a dual mechanism. On the one hand, it enhanced oxidative phosphorylation (OXPHOS) and glutaminolysis to fuel DNA repair; On the other hand, lactate accumulation drives myeloid-derived suppressor cell (MDSC) infiltration, forming a metabolic-immunosuppressive cycle (6, 7). Accumulated evidence suggests that metabolic kinases pyruvate kinase M2 (PKM2) and glucose transporter 1 (GLUT1) participate in hypoxia adaptation and programmed death-ligand 1 (PD-L1) regulation, suggesting that the metabolic network may serve as a critical nexus between radiotherapy effects and immune microenvironment remodeling (8–10). However, current research predominantly focuses on isolated pathway analyses, lacking comprehensive understanding of the systemic metabolic interaction networks underlying radiotherapy response.

Transcriptomics and single-cell metabolic analysis play pivotal roles in metabolic research. Conventional approaches such as scMetabolism primarily rely on pathway-based scoring systems. While these methods can identify alterations at the pathway level (e.g., upregulation of fatty acid metabolic pathways), they fail to provide precise information about specific metabolite transformations. This limitation stems from the inherent low resolution of pathway scoring, and the scarcity of publicly available single-cell metabolomics data. In contrast, single-cell flux estimation analysis (scFEA) employs an innovative computational framework to directly infer cell-specific metabolic flux from single-cell transcriptomic data, enabling higher-resolution metabolic state characterization. scFEA utilizes a systematically reconstructed human metabolic map as a factor graph, integrating probabilistic modeling with gradient descent algorithms to accurately trace metabolite conversion pathways. In glutamine metabolism studies, scFEA not only identified flux variations in the glutamate-to-glutathione (Glu-GSH) pathway but also demonstrated that this flux-rather than the glutamate-to-2-oxoglutarate conversion-plays a critical role in modulating tumor immune response (11). Notably, in validation experiments with matched metabolomics data, scFEA-predicted flux changes showed significant concordance with experimentally measured metabolite abundance alterations, providing empirical support for its reliability in studying metabolic heterogeneity.

This study innovatively proposed that systematic analysis of radioresistance-related metabolic genes (RRMGs) could untangle the molecular determinants of radiotherapy response. Through the synthesis and construction of an RRMG-based prognostic signature model, we have achieved clinical translational value across three dimensions for the first time: (1) In prognostic prediction, it overcomes the limitations of the conventional TNM staging system; (2) In immune microenvironment characterization, it reveals novel mechanisms of metabolism-reprogramming-mediated immunosuppression; (3) In precision therapy, it establishes a drug sensitivity prediction model. By systematically elucidating the metabolic interaction network between tumor cells and the microenvironment, this study provides a novel biomarker system and radiosensitization strategy for precision radiotherapy in NSCLC, advancing the transformation of radiation oncology toward an integrated multi-omics precision medicine paradigm.

## 2 Materials and methods

### 2.1 Data acquisition and processing

We retrieved the GSE197236, GSE157881, and GSE239514 datasets from the Gene Expression Omnibus (GEO) database (https://www.ncbi.nlm.nih.gov/geo/) for scFEA. Data from NSCLC patients was obtained from The Cancer Genome Atlas (TCGA, https://cancergenome.nih.gov/=TCGA), including bulk RNA-seq and clinical information (such as overall survival (OS), progression-free survival (PFS), treatment response, gender, age, and stage). Ultimately, 51 NSCLC samples that had undergone radiotherapy alone and complete available clinical data were included. As a validation cohort, RNA-seq data, treatment response, and survival information from the GEO database for NSCLC patients who had received combined radiotherapy and immunotherapy (GSE253564) were included.

### 2.2 Identification of co-expressed metabolic modules associated with tumor radiotherapy

Weighted gene co-expression network analysis (WGCNA) is a systems biology approach used to characterize patterns of gene relationships within samples. By leveraging central genes or module eigengenes, WGCNA can identify tightly correlated gene clusters and link these clusters to external sample traits. Based on the TCGA-LUAD dataset, a co-expression network was constructed using the WGCNA method. Through the dynamic tree-cutting algorithm, 25 gene modules were identified, and the module eigengenes (MEs) were calculated for each module. Module-trait association analysis revealed three modules significantly correlated with radioresistance (correlation coefficient r > 0.45, p < 0.01), whose module eigengenes exhibited strong associations with the radiotherapy response phenotype. Subsequently, the genes from these three modules were intersected with the KEGG metabolic pathway gene sets, ultimately yielding RRMGs.

### 2.3 Development of the prognostic model based on RRMGs

Initially, we employed univariate Cox regression and stepwise multivariate Cox regression analyses to determine the relationship between RRMGs and patient survival status. Genes with *P* < 0.05 were considered significantly associated with survival. The final set of RRMGs was used to construct a prognostic risk model. The risk score was calculated using the following formula: Risk score =∑(expGene*i* × *βi*), where expGenei is the relative expression of the prognostic model gene, and βi is the regression coeffecient.

### 2.4 Assessment and validation of the RRMGs prognostic Model

Based on the median risk score, patients were stratified into high-risk and low-risk groups. OS was evaluated using Kaplan-Meier analysis, and patient survival status was visualized according to risk scores using the “pheatmap” package in R software. Receiver operating characteristic (ROC) curve analysis and area under the curve (AUC) calculation were performed with the “timeROC” package in R. Nomograms and linear charts were constructed by integrating clinical characteristics and risk level to predict 1-year, 3-year, and 5-year overall survival rates in lung adenocarcinoma (LUAD) patients. Calibration curves were generated using the “rms,” “regplot,” and “survival” packages in R, with closer proximity to the diagonal indicating higher predictive accuracy. Furthermore, the prognostic and therapeutic efficacy predictive capability of the RRMGs model was assessed in validation cohorts GSE68465 receiving chemoradiotherapy and GSE253564 receiving combined radiotherapy and immunotherapy.

### 2.5 Analysis of immune microenvironment infiltration and immunotherapy response in risk subgroups

The CIBERSORT algorithm was employed to evaluate differences in immune infiltration between high- and low-risk groups by calculating the proportions of 22 immune cell subtypes and comparing their distribution patterns across risk subgroups. Concurrently, the Tumor Immune Dysfunction and Exclusion (TIDE) method was applied to predict individual response probabilities to immunotherapy, reflecting the potential efficacy of immune checkpoint blockers (ICBs). Wilcoxon rank-sum tests (P < 0.05) were performed to validate statistically significant differences between risk subgroups regarding immune cell infiltration fractions, TIDE scores, and immune checkpoint gene expression. Further analyses were conducted to examine differential expression levels of immune checkpoints between the two risk subtypes. The correlation between TIDE scores and immune checkpoint expression was assessed to elucidate the mechanistic association wherein the low-risk group exhibited higher immune checkpoint expression levels and lower TIDE.

### 2.6 Identification of candidate drugs for high-risk NSCLC patients

The drug sensitivity of different risk groups was evaluated using the oncoPredict R package. This tool integrates large-scale gene expression data (e.g., GDSC and CTRP databases) with drug screening data to construct a ridge regression model for predicting drug responses. In this study, the model was applied to the TCGA-LUAD gene expression dataset for drug response prediction. Further validation of the biological relevance of the predictions was performed through immune infiltration analysis (e.g., CIBERSORT, ssGSEA) and tumor microenvironment assessment (e.g., ESTIMATE algorithm). The screening criteria were set as follows: drugs with a log2 fold change (log2FC) > 0.15 and a P-value < 0.05 were considered potential therapeutic candidates. Their clinical prognostic value was additionally validated via survival analysis and risk modeling (e.g., LASSO-Cox regression). All analytical parameters, including batch effect correction using the ‘combat’ method and repeated calculation of gene expression means, were set to default values.

### 2.7 scRNA-seq data processing and analysis

Quality assessment of scRNA-seq (single-cell RNA sequencing) data were performed using FastQC software. The sequencing reads were aligned to the human GRCh38 reference genome via the STAR alignment pipeline to obtain gene counts for each sample. During subsequent analysis, cell samples with fewer than 250 or more than 10,000 expressed genes were excluded. Additionally, cells exhibiting mitochondrial genome alignment rates exceeding 15% were identified as low-quality cells and subsequently removed from the analysis.

### 2.8 scFEA

scFEA leverages a comprehensively reorganized human metabolic map as a factor graph and employs multi-layer neural networks to capture the intricate information cascade from the transcriptome to the metabolome. This approach enables the direct quantification of specific metabolic pathway abundances, thereby clarifying precisely which metabolites have undergone changes. For instance, it can elucidate the transformation of glutamate to glutamine, then to GABA, and subsequently to succinate. The “scFEA” package (available at https://github.com/changwn/scFEA) were utilized to analyze the metabolic differences between radiotherapy-sensitive and radiotherapy-resistant NSCLC cells, as well as the metabolic variations in the TIME and tumor-draining lymph nodes before and after radiotherapy.

### 2.9 Cell culture

NSCLC cell lines (A549 and LLC) were obtained from the Cell Resource Center of the Chinese Academy of Sciences (Beijing, China) and authenticated using the STR profiling method. Cells were cultured in DMEM (Gibco, Carlsbad, CA, USA) supplemented with 10% fetal bovine serum (FBS, Gibco, Carlsbad, CA, USA) and maintained at 37 °C within a humidified atmosphere containing 5% CO_2_. Regular screenings for cell mycoplasma infection were carried out and all results were confirmed to be negative.

### 2.10 Development of radioresistant cell models

The radioresistant cell models were established using a calibrated 6-MV linear accelerator (RAD SOURCE RS2000-225) with a dose rate of 17.7 Gy/min. Parental A549 and LLC cells were subjected to 2 Gy per fraction, followed by 48-hour recovery in fresh complete medium before passaging. The irradiation cycle was repeated when cells re-entered logarithmic growth phase (with recorded recovery time to normal proliferation kinetics averaging 24–48 hours post-irradiation, ensuring population synchrony). This protocol was maintained through 30 fractionated doses until a cumulative dose of 60 Gy was achieved. During model establishment, clonogenic survival assays were performed to quantify radioresistance using Survival Fraction at 2 Gy (SF2) as the endpoint. Following each fraction, cells were harvested for 10-day colony formation, with SF2 defined as the ratio of surviving colonies post-2 Gy irradiation to untreated controls. The resultant resistant lines (A549R and LLCR) demonstrated significantly elevated SF2 values compared to parental cells. Clonogenic stabilization was observed upon reaching 60 Gy total dose, confirming stable phenotypic acquisition.

### 2.11 Quantitative real time polymerase chain reaction

RNA was extracted from cells using TRIzol reagent (Vazyme, Nanjing, China). RNA concentration was measured using a spectrophotometer and stored the samples at -80 °C. cDNA was synthesized using HiScript Ill RT SuperMix for qPCR (Vazyme, Nanjing, China). Bio 7500 Real-Time PCR System (Thermo Fisher Scientific, Carlsbad, CA, USA) was used to perform qPCR in the StepOne Plus real-time PCR System (Applied Biosystems). ACTB was used as the internal reference gene for normalization. The relative fold change in expression was calculated using the 2^-ΔΔCt^ method. The sequences of target primers are detailed in **Table 1**.

**Table 1:** Primer sequences of RT-qPCR.

Gene	Forward primer (5′-3′)	Reverse primer (5′-3′)
PGD_human	GTGGCCCCACATCAAGACC	GTCCCCATACTCTATCCCGTT
PGD_mouse	CTTGTTGGACACGGGTGACAT	GCTTGGAAGATCGCCTTGATG
IDH2_human	CGCCACTATGCCGACAAAAG	ACTGCCAGATAATACGGGTCA
IDH2_mouse	ATTTTGTGGTAGATCGAGCTGG	CCTCCGGCAGGGAAGTTATAC
G6PD_human	AACATCGCCTGCGTTATCCTC	ACGTCCCGGATGATCCCAA
G6PD_mouse	CTCCAATCAACTGTCGAACCA	TTGTCTCGATTCCAGATGGGG
ALDH3A1_human	TGTTCTCCAGCAACGACAAGG	AGGGCAGAGAGTGCAAGGT
ALDH3A1_mouse	AATATCAGTAGCATCGTGAACCG	GAGAGCCCCTTAATCGTGAAATC
AACS_human	GATGACTTGTACCATTGGTCCG	ACGTGAGAAGACAATTCCACTG
AACS_mouse	GTGGAATCGTCTACTCACGCA	TAAAGGGCGACTCTGTCGTTC
UPP1_human	CTGTCAGTCATGGTATGGGCA	GAGCACCGGGCATAGTACA
UPP1_mouse	TGAAGGAAGACGTGCTCTACC	GAAGGTGTTCATCCGGGAAGA
XYLT2_human	AGGTGGTACGGGCAGTAAC	GCTCCCTGTATCTCCGTGT
XYLT2_mouse	TTAAAGGACGTGGACTCGCTT	TGGGTGGAGTTAAACTGCCTC
ACTB_human	GGCTGTATTCCCCTCCATCG	CCAGTTGGTAACAATGCCATGT
ACTB_mouse	CATGTACGTTGCTATCCAGGC	CTCCTTAATGTCACGCACGAT

### 2.12 Western blot

Whole cell mixtures were separated using cell complete lysis buffer for western and IP (Beyotime, item number: P0037-100ml, Shanghai, China), protease inhibitor, and protein phosphatase inhibitor mixtures. The protein concentration was measured by using BCA protein concentration rapid determination kit (Beyotime, Shanghai, China). Equal amounts of proteins were separated on 10% SDS-PAGE and transferred to a 0.45 μm PVDF membrane (Millipore, IPFL00005, Darmstadt, Germany). The membranes were closed with 5% skimmed milk and incubated with primary antibodies, including anti-PGD (Abways, DY1193, Beijing, China), anti-IDH2 (Abways, CY7005), anti-G6PD (Abways, CY6841), anti-ALDH3A1 (Proteintech, 20874–1-AP, Wuhan, China), anti-AACS (Proteintech, 13815-1-AP) and anti-β-tubulin (Cell Signaling Technology, #2146S, Danvers, MA, USA), overnight at 4 °C. They were then incubated in secondary IgG (ABclonal, AS014, Wuhan, China) for 1 h at room temperature. Protein bands were visualized using Super ECL detection reagent (Vazyme, Nanjing, China). Grayscale analysis of protein bands was performed using Image J software.

### 2.12 Statistical methods

Statistical analysis was performed using IBM SPSS Statistics 26 and R software (4.3.3). Bootstrapping was used to avoid over fitting. The normal distribution variables were analyzed by Student’s *t*-test. Non-normally distributed variables were analyzed using Wilcoxon rank sum test. *P*<0.05 was considered statistically significant.

## 3 Results

### 3.1 Metabolic analysis of parental and radioresistant A549 cells

**Figure 1:**
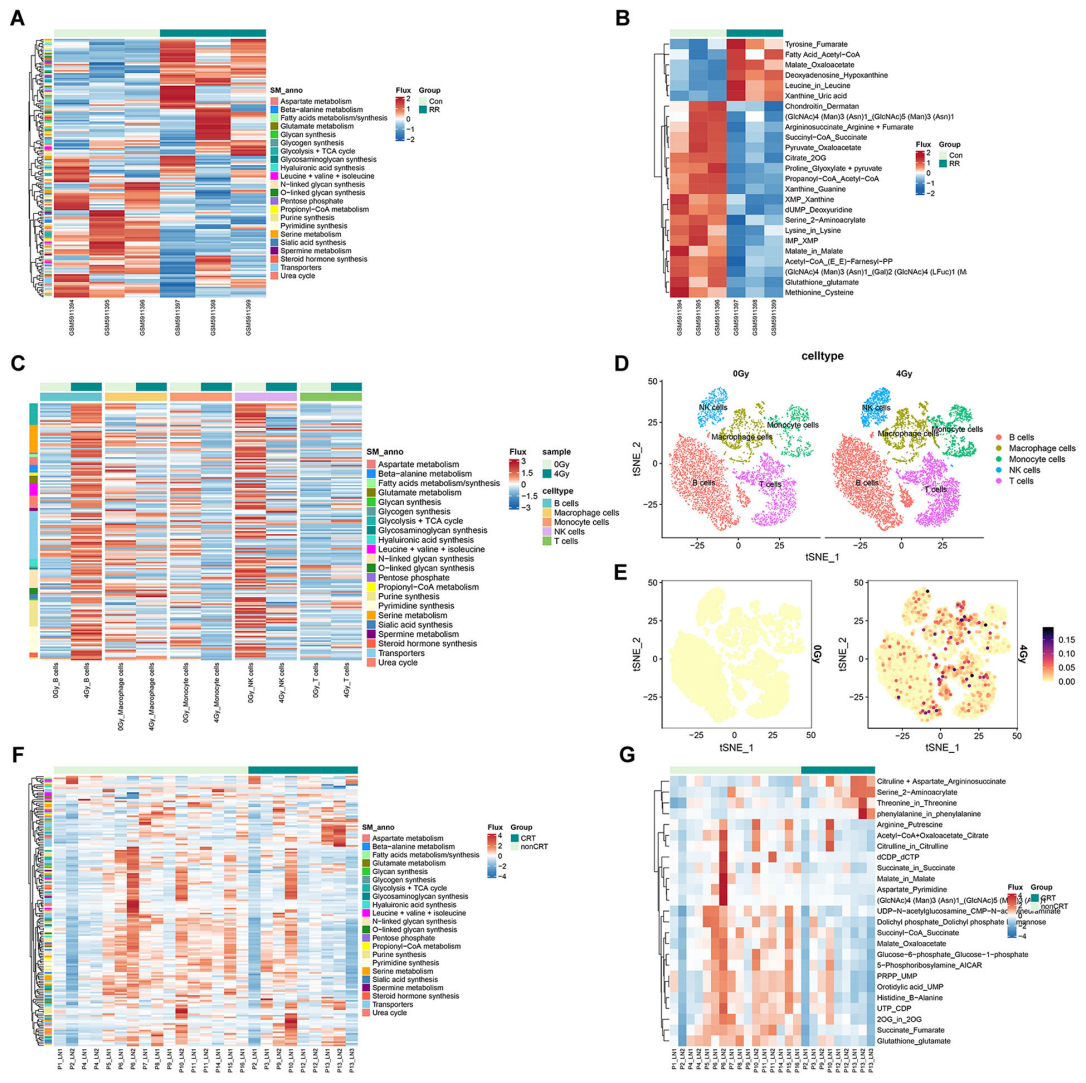
Metabolic reprogramming in the TIME associated with radiotherapy. **(A)** Overview of all 168 metabolic flux pathways (divided into 22 supermodules) in A549 parental and radioresistant cells. **(B)** Heatmap of the 25 significantly different metabolic fluxes between A549 parental and radioresistant cells (*P*<0.05). **(C)** Heatmap of metabolic fluxes in various immune cells before and after radiotherapy. **(D)** Single-cell clusters of mouse HKP1 lung orthotopic carcinoma before and after radiotherapy (3×4 Gy). **(E)** Cluster of metabolic flux changes in serine metabolism (M30 flux) in various immune cells before and after radiotherapy. **(F, G)** Overview of all 168 metabolic flux pathways (divided into 22 supermodules) in tumor-draining lymph nodes (TDLNs) from NSCLC patients with primary tumors that were untreated (nonCRT) or treated with chemoradiotherapy (CRT). Among these, 21 metabolic fluxes showed significant differences between the two groups (*P*<0.05).

Based on the bulk RNA-seq data from GSE197236, we used scFEA to transform transcriptomics into metabolomics, analyzing the metabolic differences between parental A549 cells and radioresistant A549 cells. The lung cancer cell line A549 in logarithmic growth phase was irradiated with 4 Gy for the first time and cultured for subculture. After cell adherence growth, the cells were irradiated again with 4 Gy, with a total dose of 60 Gy. **Figure 1A** shows the differences in 168 metabolic fluxes and 22 metabolic supermodules between parental and radioresistant A549 cells. There were 25 significantly altered metabolic fluxes between parental and radioresistant A549 cells, with 6 fluxes increased and 19 fluxes decreased in radioresistant cells (**Figure 1B**).

Compared with parental cells, most metabolic fluxes in radioresistant cells demonstrated a downward trend. Specifically, fluxes related to N-linked glycan synthesis, including M122 [(GlcNAc)4(Man)3(Asn)1→(Gal)2(GlcNAc)4(LFuc)1(Man)3(Neu5Ac)2(Asn)1)],M124[(GlcNAc)4(Man) 3(Asn)1→(GlcNAc)5(Man)3 (Asn)1)], and M113[(Acetyl-CoA→(E,E)-Farnesyl-PP)] were decreased. Additionally, fluxes in the TCA cycle, such as M10 (Succinyl-CoA→Succinate), M8(Citrate→2OG) and M14 (Pyruvate→Oxaloacetate) were also reduced. The intermediate metabolite (GlcNAc)4(Man)3(Asn)1 in the N-linked glycan synthesis fluxes M122 and M124 was increased. Interestingly, the enzymes regulating M122 and M124, including N-acetylglucosamine transferases (MGAT1, MGAT3, and MGAT4A), were downregulated in radioresistant A549 cells. In the TCA cycle fluxes M10 and M8, the metabolites Succinyl-CoA and Citrate were increased, while Succinate and 2OG were decreased.

For purine synthesis metabolism, fluxes related to IMP degradation and xanthine synthesis and transformation were reduced, including M137(IMP→XMP), M145 (XMP→Xanthine) and M147 (Xanthine→Guanine); while the flux of xanthine degradation M146 (Xanthine→Uric acid) was increased. The metabolite xanthine was decreased, while IMP was increased.

### 3.2 Metabolic differences in the TIME of mouse HKP1 lung orthotopic carcinoma before and after radiotherapy

Based on scRNA-seq data from the GSE157881 database, we employed the scFEA method to evaluate metabolic flux alterations in various immune cell populations within the TME and systematically compared metabolic differences before and after radiotherapy. Single live cells sorted from HKP1-bearing lungs which received 0Gy-RT or 4Gy-RT were sequenced. The mutant HKP1 (*Kras^G12D^p53*^−/−^) orthotropic mouse model of lung cancer develops adenocarcinoma with histopathological similarities to human NSCLC in immunocompetent C57/BL6 mice. **Figure 1C** demonstrates the differential metabolic fluxes across 168 metabolic pathways (22 supermodules) among distinct immune cell types pre- and post-radiotherapy. The study revealed that different immune cell subsets exhibited markedly heterogeneous metabolic responses to radiotherapy: B cells displayed the most pronounced upregulation of metabolic flux post-radiotherapy, whereas natural killer (NK) cells showed the most significant metabolic suppression. In contrast, T cells exhibited minimal changes in metabolic flux before and after radiotherapy. Despite substantial overall metabolic variations among immune cell populations, we observed that the serine metabolic flux within the methionine/cysteine metabolic pathway (M30 pathway) consistently demonstrated the most prominent enhancement across all immune cell types following radiotherapy (**Figures 1D, E**).

### 3.3 Metabolic differences in TDLNs of patients before and after radiotherapy

Based on the GSE239514 dataset (which performed bulk RNA sequencing on 25 TDLN samples from 16 NSCLC patients), we employed scFEA to transform transcriptomic data into metabolomic data. By comparing metabolic differences in TDLNs between patients who were not subjected to chemoradiotherapy (nonCRT) and those who received chemoradiotherapy (CRT), it was found that most metabolic fluxes in TDLNs exhibited a downregulating trend post-radiotherapy (**Figure 1F**). **Figure 1G** demonstrates 21 significantly altered metabolic fluxes between the two patient groups. Specifically: 1) The fluxes of malate → oxaloacetate (M13) and acetyl-CoA + oxaloacetate → citrate (M7) in the tricarboxylic acid (TCA) cycle; 2) The flux of dolichyl phosphate → dolichyl phosphate D-mannose (M118) in N-linked glycan biosynthesis; and 3) The flux of aspartate → pyrimidine (M36) in aspartate metabolism were all significantly reduced. Notably, in the M7 pathway, increased levels of acetyl-CoA and oxaloacetate were observed alongside decreased citrate, while in the M118 pathway, elevated dolichyl phosphate was accompanied by reduced dolichyl phosphate D-mannose. These results indicate that radiotherapy can induce aberrant expression of metabolic enzymes and accumulation of intermediate metabolites in the NSCLC tumor microenvironment (including tumor cells, TIME, and local TDLNs), thereby triggering metabolic reprogramming and mediating radioresistance.

### 3.4 Identification of metabolic genes associated with radioresistance

**Figure 2:**
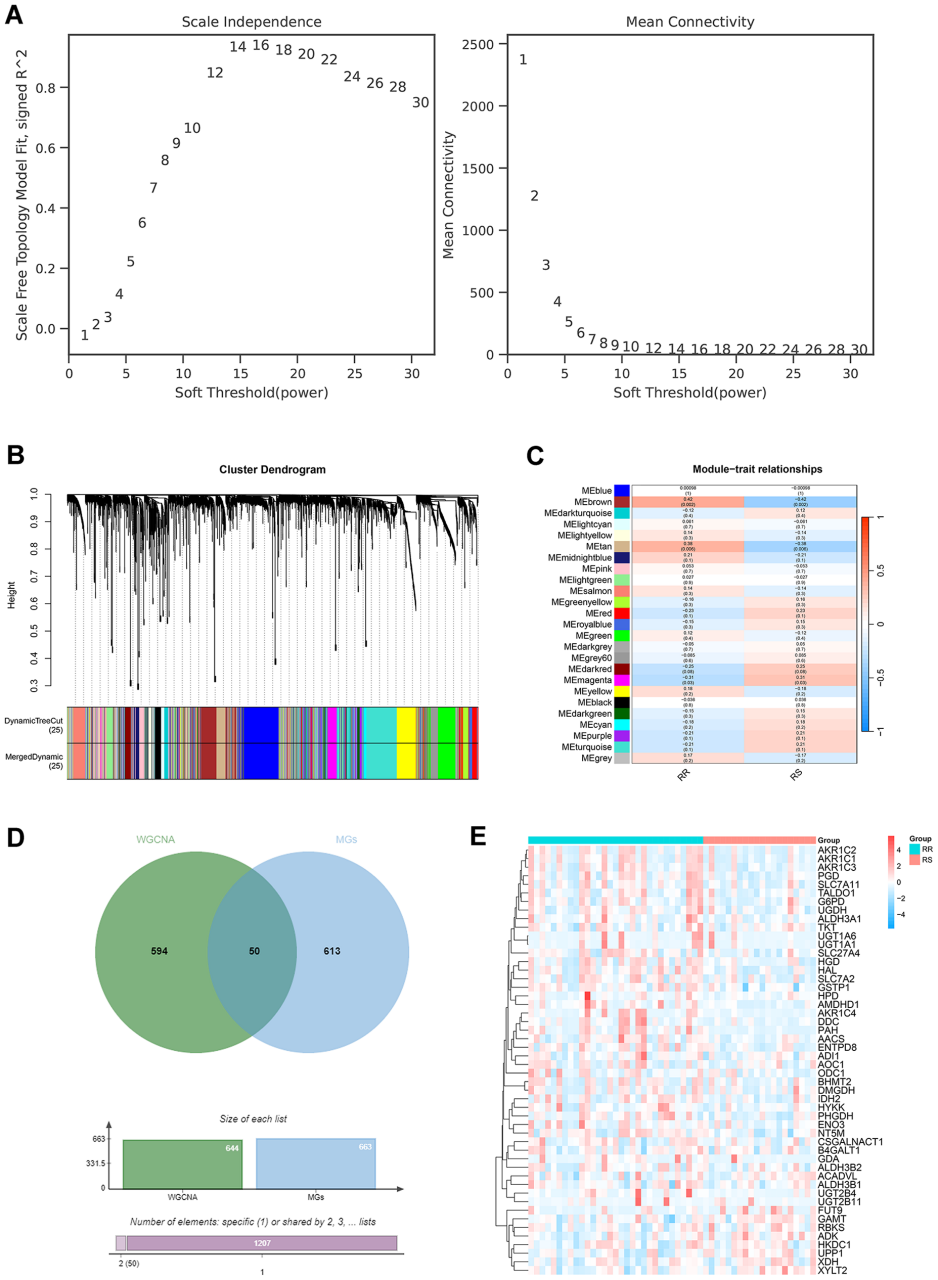
Identification of co-expression gene modules associated with radioresistance. **(A)** Selection of soft threshold based on scale-free topology. **(B)** Selection of soft threshold based on average connectivity. **(C)** Dendrogram of genes associated with radiotherapy. **(D)** Heatmap of the correlation between gene co-expression modules and radiotherapy response. **(E)** Venn diagram illustrating the selection of radioresistance-related metabolic genes (RRMGs). **(F)** Heatmap showing the expression levels of RRMGs in the RR group and the RS group.

Based on the TCGA database, we obtained data from 554 LUAD patients, and ultimately selected 51 patients who had received radiotherapy alone. The RNA-seq data from these patients included 11,105 genes. After filtering out genes with low expression variability (standard deviation ≤ 0.5), 4,570 genes remained. These genes were then subjected to WGCNA. First, the optimal soft threshold power for a scale-free network was calculated to be 12, with a correlation coefficient greater than 0.8 (**Figure 2A**). Subsequently, 25 modules were identified based on average linkage hierarchical clustering and the optimal soft threshold power (**Figure 2B**). Those patients selected from the TCGA database were divided into radioresistant (RR) and radiosensitive (RS) groups. RR was defined as complete remission (CR) after radiotherapy, while other types (such as stable disease (SD) and progressive disease (PD)) were classified as RS. Correlation analysis was conducted between the 25 modules and radiotherapy, revealing that MEbrown (343 genes) and MEtan (150 genes) were positively correlated with RR, while MEmagenta (153 genes) was negatively correlated with RR (**Figure 2C**). The WGCNA module gene sets associated with radioresistance included MEbrown, MEmagenta, and MEtan, comprising 644 genes. The metabolic-related gene set consisted of 663 genes, derived from all metabolic-related genes in the 168 metabolic pathways identified in the first part using scFEA. The intersection of the radioresistance-associated WGCNA module gene sets and the metabolic-related genes yielded 50 genes, which were used for further analysis (**Figures 2D, E**).

### 3.5 Development and validation of the radiotherapy prognostic model

**Figure 3:**
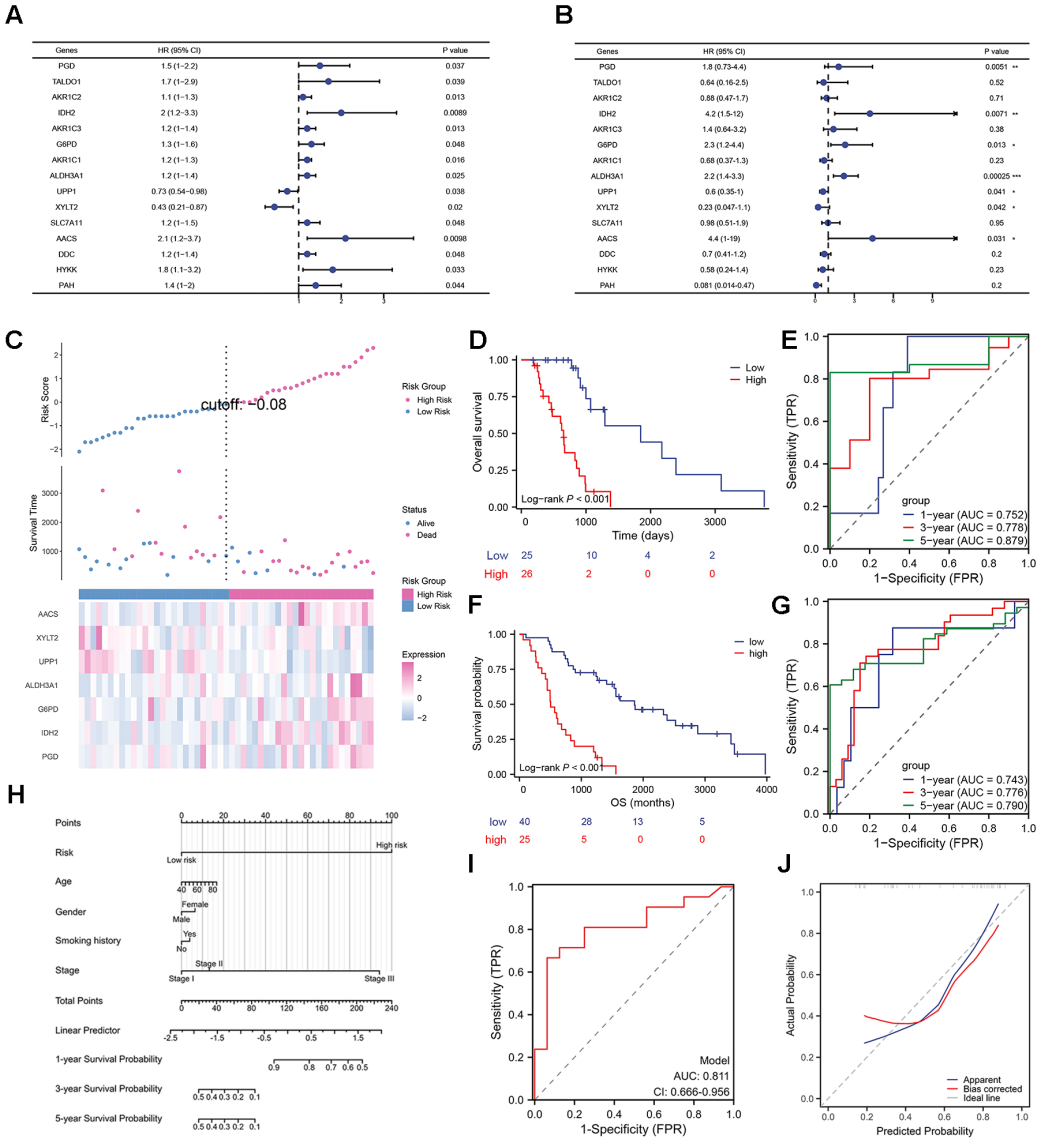
Construction and evaluation of the radiotherapy prognostic model. **(A, B)** Forest plots of univariate and multivariate Cox regression analyses for survival-related genes. **(C)** Risk factor plot, with the upper panel showing the stratification of patients into high- and low-risk groups based on risk scores, the middle panel displaying survival status in these two groups, and the lower panel further analyzing the differences in RRMGs expression across different risk groups. **(D, F)** OS curves of risk subgroups for TCGA-LUAD and GSE68465 dataset. **(E, G)** Time-dependent ROC curves and area under the curve (AUC) values of the RRMG-prognostic model for TCGA-LUAD and GSE68465 dataset. **(H)** Nomogram constructed by integrating risk scores and clinical features. **(I, J)** Predictive ROC curves and calibration curves for the nomogram. *P<0.05, **P<0.01, ***P<0.001.

To investigate the prognostic value of RRMGs, we initially performed univariate Cox regression analysis (stratified by RR and RS subgroups) on 50 candidate genes, identifying 15 genes significantly associated with survival. Subsequent stepwise Cox regression analysis ultimately identified seven independent prognostic biomarkers: phosphogluconate dehydrogenase (PGD), isocitrate dehydrogenase 2 (IDH2), glucose-6-phosphate dehydrogenase (G6PD), aldehyde dehydrogenase 3A1 (ALDH3A1), uridine phosphorylase 1 (UPP1), xylosyltransferase 2 (XYLT2), and acetyl-CoA synthetase (AACS). Among these, PGD, IDH2, G6PD, ALDH3A1, and AACS were determined as risk factors, whereas UPP1 and XYLT2 exhibited protective effects (**Figures 3A, B**).

Based on these seven key RRMGs, a multivariate Cox regression prognostic model was constructed. The risk score was calculated as follows: Risk score = (0.3153 × PGD expression) + (0.7059 × IDH2 expression) + (0.4029 × G6PD expression) + (0.4784 × ALDH3A1 expression) + (0.8238 × AACS expression) - (0.3972 × UPP1 expression) - (0.3711 × XYLT2 expression). Patients with LUAD were stratified into high- and low-risk groups based on the median risk score. Kaplan-Meier analysis demonstrated significantly shorter overall survival in the high-risk group compared to the low-risk group (HR = 4.726 [95% CI: 2.154-10.371], p < 0.001) (**Figure 3D**). The AUC values for 1-, 3-, and 5-year follow-up were 0.752, 0.778, and 0.879, respectively (**Figure 3E**).

In addition, the prognostic performance of our RRMGs model was validated using the independent dataset GSE68465 (**Figures 3F, G**). This dataset originated from a large, multi-institutional, blinded validation study that evaluated multiple gene expression-based prognostic models in 442 lung adenocarcinoma cases. From this cohort, we selected 65 patients who received radiotherapy and had complete clinical and survival records for further validation. Kaplan-Meier analysis revealed significantly worse overall survival in the high-risk group compared to the low-risk group (HR = 4.163, 95% CI: 2.025-8.558, P < 0.001; **Figure 3F**). The RRMGs model demonstrated strong predictive accuracy with 1-, 3-, and 5-year AUC values of 0.743, 0.776, and 0.790, respectively, in the GSE68465 dataset (**Figure 3G**). These results confirm the robust prognostic capability of our model.

Finally, the RRMGs prognostic model was integrated with clinical parameters (age, gender, smoking history and stage) to construct a prognostic nomogram (**Figure 3H**). ROC curve showed that this prognostic model exhibited excellent predictive performance with good probability (AUC = 0.811, **Figures 3I, J**).

### 3.6 Analysis of tumor immune microenvironment infiltration, immune therapy sensitivity, and prognosis in radioimmunotherapy

**Figure 4:**
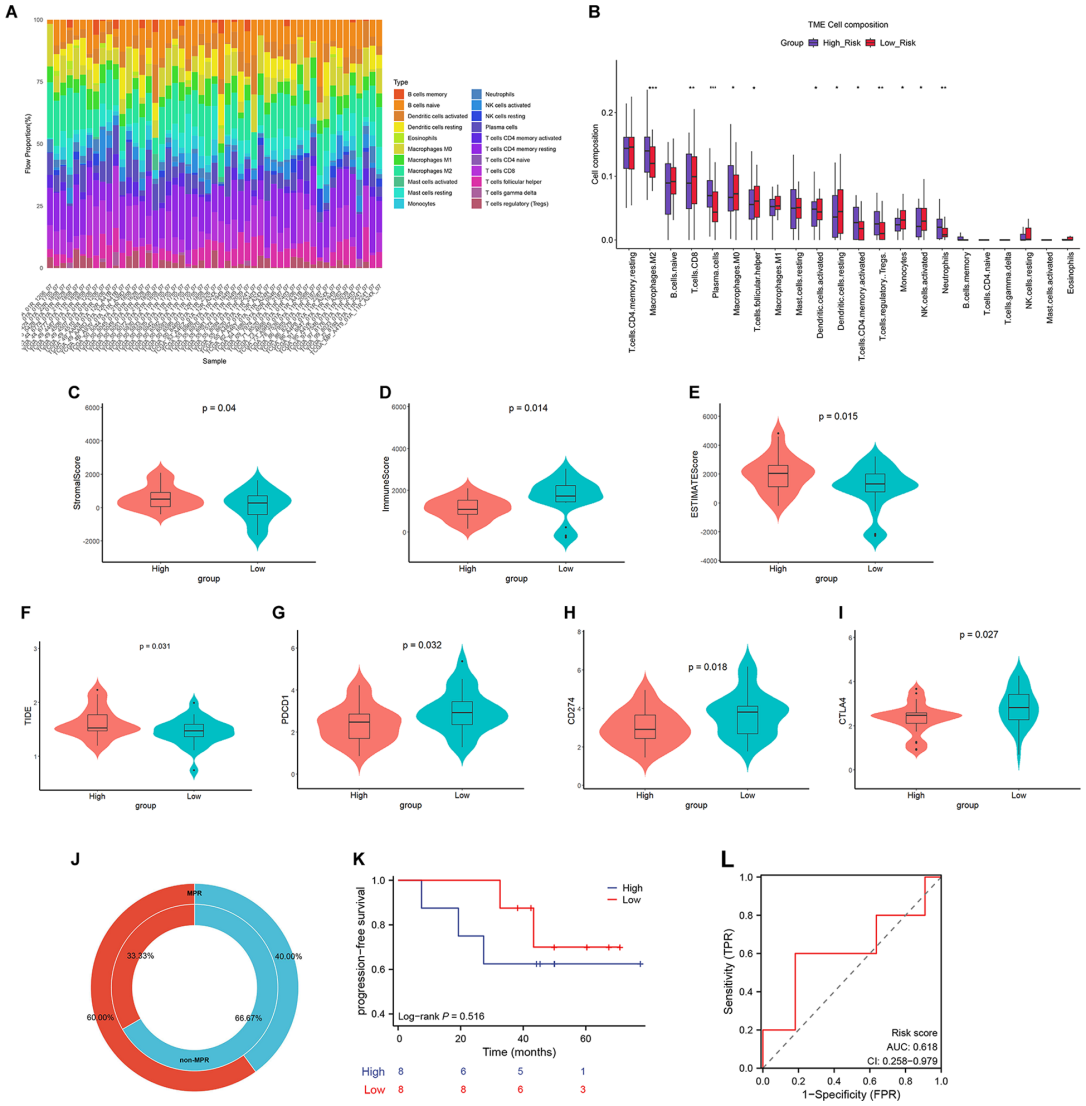
Tumor immune microenvironment, immune therapy sensitivity, and prognosis of the RRMG model in radioimmunotherapy. **(A)** Distribution of immune cells in LUAD patients receiving radiotherapy. **(B)** Immune cell infiltration levels in different risk subgroups of LUAD patients. **(C–E)** ESTIMATE analysis metrics (Stromal Score, Immune Score, and ESTIMATE Score) in different risk subgroups. **(F)** TIDE scores in different risk subgroups. **(G–I)** Expression of immune checkpoints *PDCD1*, *CD274*, and *CTLA4* in different risk subgroups. **(J)** Donut chart showing the proportion of high- and low-risk groups in MRP and non-MRP. **(K)** PFS curves for high- and low-risk groups. **(L)** ROC curves and AUC values of the RRMG prognostic model. *P<0.05, **P<0.01, ***P<0.001.

Given that radiotherapy can remodel the tumor microenvironment (TME) to either enhance or suppress anti-tumor immune responses, we employed the CIBERSORT algorithm to analyze immune cell infiltration patterns across risk subgroups. Significant differences in infiltration levels of various immune cell types were observed (**Figures 4A, B**). Specifically, the high-risk group exhibited reduced infiltration of anti-tumor immune cells such as CD8+ T lymphocytes and natural killer (NK) cells, while showing increased infiltration of pro-tumor cells including M2 macrophages, regulatory T cells (Tregs), and neutrophils. ESTIMATE algorithm analysis revealed elevated Stromal Scores and ESTIMATE Scores but decreased ImmuneScores in the high-risk group (**Figures 4C–E**), indicating an immunosuppressive TME phenotype. To investigate differential responses to immunotherapy among risk subgroups, we predicted treatment efficacy using the TIDE algorithm (where higher TIDE scores suggest greater likelihood of immune evasion). The high-risk group demonstrated significantly higher TIDE scores (**Figure 4F**). Furthermore, we examined expression differences of immune checkpoint molecules - programmed cell death protein 1 (PDCD1), programmed death-ligand 1 (CD274), and cytotoxic T-lymphocyte-associated protein 4 (CTLA4) - across subgroups (**Figures 4G–I**). Downregulation of these immune checkpoint molecules was observed in the high-risk group, suggesting potential immune evasion mechanisms promoting tumor progression.

RNA-seq data from 16 patients in the GSE253564 dataset who underwent combined radiotherapy and immunotherapy were analyzed. Patients were stratified by pathological response into major pathological response (MPR; >90% tumor regression, n=10) and non-MPR (≤90% regression, n=6) groups. The MPR group predominantly comprised low-risk patients, whereas the non-MPR group consisted mainly of high-risk cases (**Figure 4J**). Kaplan-Meier survival analysis and ROC curve evaluation confirmed poorer prognosis in the high-risk group although there was no significant difference (P = 0.516, AUC = 0.618; **Figures 4K, L**). These results demonstrate that the RRMG prognostic model not only effectively predicts outcomes for radiotherapy alone but also shows significant value in predicting therapeutic efficacy for combined radiotherapy and immunotherapy.

### 3.7 High-risk RRMGs were upregulated in radioresistant NSCLC cells

**Figure 5:**
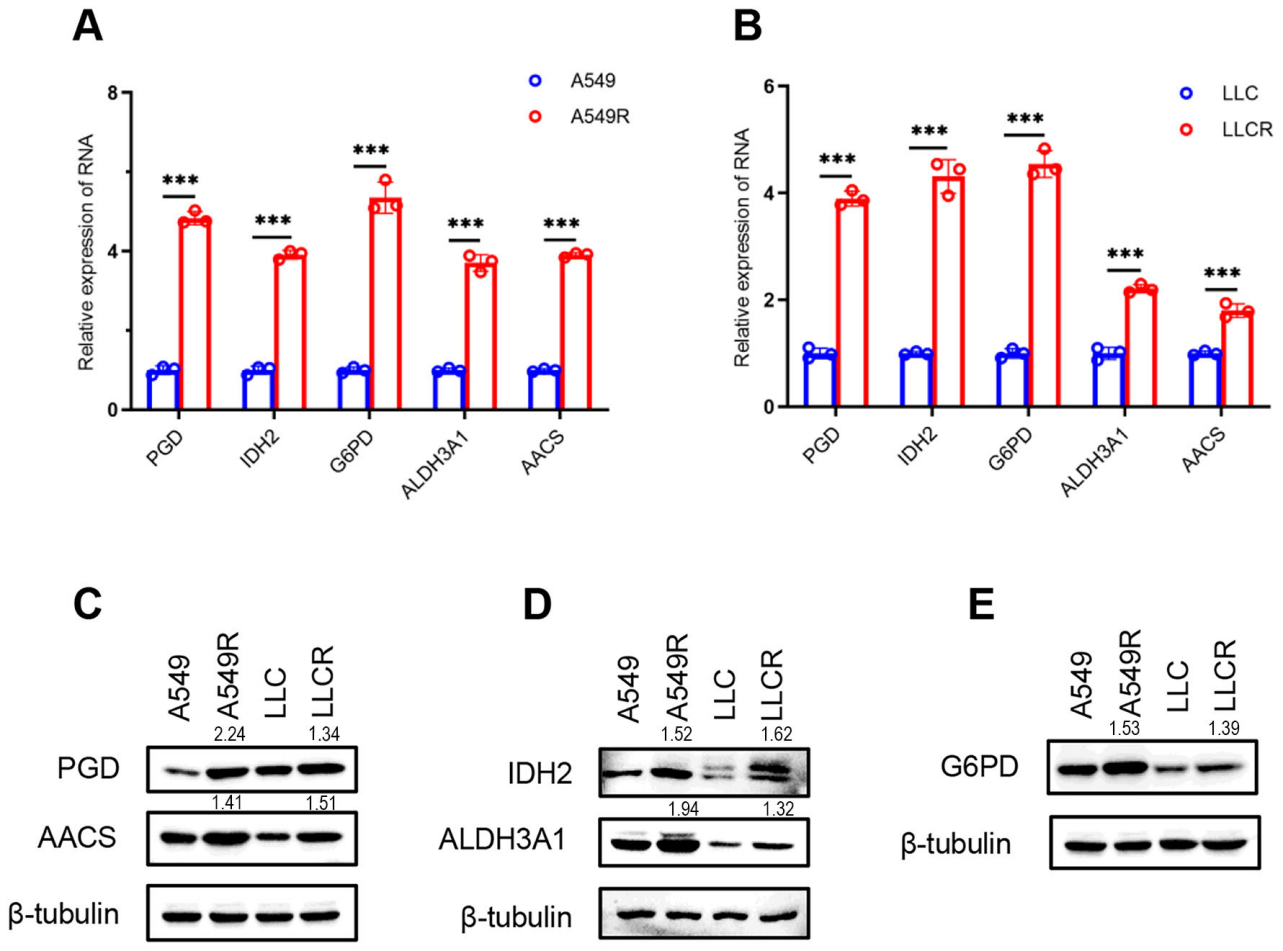
Experimental validation of high-risk RRMGs in parental and radioresistant NSCLC cells. **(A, B)** RNA expression levels of high-risk RRMGs in parental (A549 and LLC) and radioresistant NSCLC cells (A549R and LLCR). **(C–E)** Protein expression levels of high-risk RRMGs in parental and radioresistant NSCLC cells. ***P<0.001.

To investigate the expression profiles of high-risk RRMGs in radioresistant NSCLC cells, radiation-resistant cell lines A549R and LLCR were successfully established by subjecting A549 and LLC cells to fractionated irradiation with a cumulative dose of 60 Gy. qRT-PCR and Western blot analyses demonstrated that both mRNA and protein expression levels of PGD, IDH2, G6PD, ALDH3A1, and AACS were significantly upregulated in the radioresistant cells (**Figures 5A–E**). These findings not only validate the reliability of our analytical results but also suggest that these genes may play a critical regulatory role in the development of radioresistance in NSCLC.

### 3.8 Identification and analysis of candidate drugs for high-risk patients

**Figure 6:**
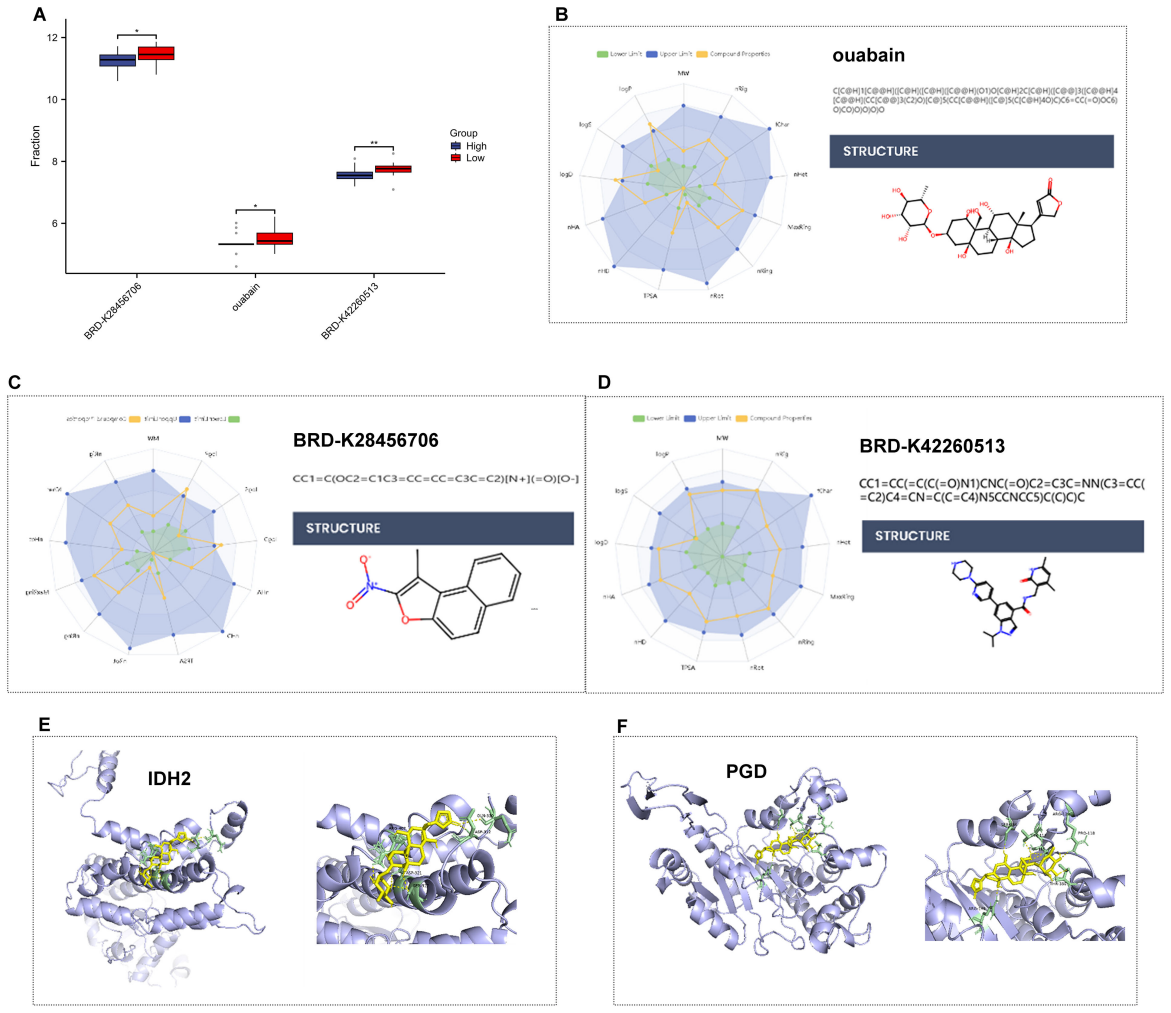
Candidate radio-immunotherapy sensitizers for high-risk patients. **(A)** Drug response results for potential therapeutic agents (BRD-K42260513, ouabain, and BRD-K28456706) across different risk subgroups. **(B–D)** Molecular structures of the three potential therapeutic agents (BRD-K42260513, ouabain, and BRD-K28456706). **(E)** Predicted 3D docking models of IDH2 with the compound ouabain. **(F)** Predicted 3D docking models of PGD with the compound ouabain. *P<0.05, **P<0.01.

To identify potential radiosensitizers and immunotherapy sensitizers applicable to high-risk patients, we evaluated differences in drug sensitivity among distinct risk subgroups based on drug response data predicted by the “oncoPredict” R package. By screening for drugs exhibiting higher sensitivity in the high-risk population, differential drug response analysis was first performed to identify compounds with significant differences, retaining only those with lower half-maximal inhibitory concentration (IC50) estimates in the high-risk group. Three drugs/compounds demonstrating potential therapeutic value for high-risk patients were ultimately identified: BRD-K42260513, ouabain, and BRD-K28456706 (**Figures 6A–D**). Analysis of absorption, distribution, metabolism, excretion, and toxicity (ADMET) properties revealed that ouabain exhibited the most favorable overall profile, followed by BRD-K28456706 and BRD-K42260513 (**Table 2**). For in-depth analysis of these three candidates, molecular docking was performed between five high-risk radioresistance-associated metabolic proteins (PGD, IDH2, G6PD, ALDH3A1, and AACS) and the candidate drugs. **Figures 6E, F** illustrate the docking models of PGD and IDH2 with ouabain, demonstrating binding free energies of -7.9 kcal/mol and -8.7 kcal/mol, respectively. These candidate drugs show promise asradiosensitizers or radioimmunotherapy sensitizers for NSCLC treatment.

**Table 2:** ADMET analysis of three candidate radiosensitizers.

Compounds	Absorption	Distribution	Metabolism	Excretion	Toxicity
	Caco-2	PPB	CYP3A4	CL	HERG
BRD-K28456706	-4.621	98.713	0.377	4.752	0.021
ouabain	-7.045	28.077	0	1.064	0.595
BRD-K42260513	-5.624	78.05	0.992	6.164	0.531

## 4 Discussion

This study systematically untangled the metabolic reprogramming characteristics of radioresistant lung cancer cells and their dynamic changes within the tumor microenvironment through multi-omics analysis, providing crucial evidence for understanding radioresistance mechanisms and developing novel metabolic intervention strategies. By systematically analyzing the expression profiles of RRMGs in LUAD, we successfully established and validated a prognostic prediction model based on seven key RRMGs (PGD, IDH2, G6PD, ALDH3A1, AACS, UPP1, and XYLT2). This model not only effectively distinguished radiotherapy sensitivity among LUAD patients but also predicted clinical outcomes of combined radiotherapy and immunotherapy, offering a novel molecular tool for personalized treatment decision-making.

There is currently a lack of comprehensive single-cell metabolomics and TME analyses related to radiotherapy in NSCLC. Therefore, we utilized scFEA to analyze the metabolic reprogramming differences in NSCLC tumor cells, immune microenvironment, and tumor-draining lymph nodes based on bulk and single-cell RNA-seq. We found that the metabolic changes in these three areas are heterogeneous, allowing us to more systematically and comprehensively understand the radiotherapy-related metabolic reprogramming in the NSCLC TME. This approach overcomes the limitations of traditional analyses that focus solely on specific metabolic pathways or metabolites in tumor cells or certain immune cells.

In this study, the suppression of the IMP degradation pathway (M137, M145) in radioresistant cells was accompanied by enhanced xanthine/uric acid conversion, which aligns with the hypothesis that tumor cells remodel purine metabolism to regulate DNA damage repair and inhibiting purine synthesis enhances radiosensitivity, supporting the potential therapeutic implications of the metabolic signatures identified in this study (12, 13). Single-cell analysis uncovered significant heterogeneity in the metabolic responses of immune cell subsets following radiotherapy. Notably, all immune cell types demonstrated activation of the methionine/cysteine pathway (M30), suggesting that radiotherapy may induce oxidative stress, thereby forcing immune cells to upregulate antioxidant metabolism (14). Collectively, these findings support the hypothesis that dynamic changes in tumor microenvironment metabolites may serve as predictive biomarkers for radiotherapy response (15, 16).

The study revealed significant upregulation of high-risk genes including PGD, IDH2, G6PD, ALDH3A1, and AACS in radioresistant cell lines, which aligns with previous findings demonstrating the close association between metabolic reprogramming and radiotherapy resistance (17, 18). Notably, G6PD and IDH2—key enzymes in the pentose phosphate pathway and TCA cycle respectively—may protect tumor cells from radiation-induced oxidative damage through enhanced NADPH production and antioxidant capacity (19). As a member of the aldehyde dehydrogenase family, ALDH3A1 has been associated with cancer stem cell properties and chemoresistance, and this study is the first to confirm its role in radioresistance (20). Of particular interest, the low expression of protective genes UPP1 and XYLT2 may enhance radiosensitivity by affecting pyrimidine metabolism and extracellular matrix remodeling suggesting potential targets for developing novel radiosensitizers (21, 22).

The multigene prognostic model developed in this study demonstrated superior predictive performance in both the development set and validation set (with 1-year, 3-year, and 5-year AUC values of 0.752, 0.778, and 0.879, respectively), significantly outperforming traditional clinical staging systems. These findings align with recent studies on LUAD prognosis based on metabolic genes (23–25), while the novelty of this work lies in its pioneering association between metabolic characteristics and radiotherapy response specificity. The predictive performance was further enhanced when clinical parameters were integrated into the nomogram, consistent with the current precision medicine paradigm emphasizing multidimensional assessment (26, 27). Notably, this model showed potential value in predicting outcomes of combined radiotherapy and immunotherapy, as evidenced by the predominance of low-risk patients in the MPR group, suggesting that metabolic features may influence immunotherapy response (28, 29). Although the RRMGs model achieved an AUC of 0.618 in the radioimmunotherapy cohort, it remained significantly associated with immune escape phenotypes and trends in treatment response, indicating that metabolic features retain biological relevance in the combined therapy setting. Given the added complexity introduced by immunotherapy, a metabolism-focused model may have limited predictive power for combination treatment outcomes. Future studies could explore integrating metabolic and immune signatures to develop a more comprehensive prognostic model for improved predictive accuracy.

Our study revealed that high-risk patients exhibited distinct features of an inhospitable environment. These observations corroborate the theoretical framework of metabolic reprogramming shaping an immunosuppressive tumor microenvironment (TME) (30, 31). ESTIMATE analysis demonstrated high stromal scores and low immune scores, potentially indicating cancer-associated fibroblast (CAF) activation and immune cell exclusion (32). The low expression of immune checkpoint molecules PD-1/PD-L1 and CTLA-4 in the high-risk group implies the existence of non-classical immune evasion mechanisms (33, 34), providing new insights into metabolism-immune interactions. The differential TIDE scores further confirmed that high-risk patients were more prone to immune evasion (35, 36), suggesting that these patients might benefit from combined metabolic intervention and immunotherapy.

Drug repurposing, which involves identifying new therapeutic targets for existing drugs, has gained increasing attention in the development of treatments for various cancers, neurological disorders, and infectious diseases. Our research identified candidate drugs (ouabain, BRD-K42260513, and BRD-K28456706) from pharmacogenomic databases that showed enhanced therapeutic sensitivity in high-risk groups. Ouabain, a cardiac glycoside, inhibits the Na+/K+-ATPase pump, increasing intracellular calcium levels and activating multiple immune-related signaling pathways. This compound has been shown to induce apoptosis and inhibit proliferation in various cancer cell lines, including breast, prostate, melanoma, pancreatic, lung, leukemia, neuroblastoma, and renal cancers. Additionally, it has been reported to enhance radiosensitivity in prostate, cervical, and glioblastoma cancers (37, 38). While information on BRD-K28456706 and BRD-K42260513 is limited, future studies will further elucidate their potential value in radio-immunotherapy for NSCLC.

This study has the following limitations. (1) The ex vivo radioresistance model may not fully recapitulate the complexity of the tumor microenvironment *in vivo*. (2) The metabolic flux analysis was based on computational predictions and requires further experimental validation. (3) This study represents an exploratory investigation of biomarkers and prognostic modeling. The developed RRMGs model would require prospective, multi-center clinical trials to rigorously evaluate its clinical utility in real-world decision-making. (4) This study was developed using a lung adenocarcinoma (LUAD) cohort and has not yet been validated in squamous cell carcinoma or other NSCLC subtypes. Given the distinct metabolic reprogramming patterns, immune microenvironment characteristics, and treatment responses across different histological subtypes, the generalizability of the RRMGs model beyond LUAD remains uncertain. Future studies should assess its predictive performance and biological relevance in independent cohorts of additional NSCLC subtypes, particularly lung squamous cell carcinoma. (5) The specific molecular mechanisms by which RRMGs (radioresistance-related molecular groups) regulate radiotherapy resistance remain to be elucidated through ex vivo experiments. (6) While our computational drug screening identified promising therapeutic candidates for high-risk patients, the radiosensitizing effects of these compounds require further experimental validation.

## 5 Conclusion

In conclusion, we have developed a promising RRGM-based prognostic model for predicting therapeutic efficacy and outcomes of radioimmunotherapy in NSCLC, while simultaneously characterizing the tumor immune microenvironment. Furthermore, we have identified three potential candidate drugs (ouabain, BRD-K28456706, and BRD-K42260513) and conducted a systematic analysis of TME metabolic features, providing preliminary insights for stratification and personalized treatment strategies in NSCLC patients.

## Data Availability

The original contributions presented in the study are included in the article/supplementary material. Further inquiries can be directed to the corresponding authors.
